# Oral epithelial cell sheets engraftment for esophageal strictures after endoscopic submucosal dissection of squamous cell carcinoma and airplane transportation

**DOI:** 10.1038/s41598-017-17663-w

**Published:** 2017-12-12

**Authors:** Naoyuki Yamaguchi, Hajime Isomoto, Shinichiro Kobayashi, Nobuo Kanai, Kengo Kanetaka, Yusuke Sakai, Yoshiyuki Kasai, Ryo Takagi, Takeshi Ohki, Hiroko Fukuda, Tsutomu Kanda, Kazuhiro Nagai, Izumi Asahina, Kazuhiko Nakao, Masayuki Yamato, Teruo Okano, Susumu Eguchi

**Affiliations:** 1 Department of Gastroenterology and Hepatology, Nagasaki University Hospital, Nagasaki, Japan; 2 Division of Medicine and Clinical Science, Department of Multidisciplinary Internal Medicine, Tottori University Faculty of Medicine, Yonago, Japan; 3 Department of Surgery, Nagasaki University Graduate School of Biomedical Sciences, Nagasaki, Japan; 4 Institute of Advanced Biomedical Engineering and Science, Tokyo Women’s Medical University, Tokyo, Japan; 5 Department of Surgery, Institute of Gastroenterology, Tokyo Women’s Medical University, Tokyo, Japan; 6 Transfusion and Cell Therapy Unit, Nagasaki University Hospital, Nagasaki, Japan; 7 Department of Regenerative Oral Surgery, Nagasaki University Graduate School of Biomedical Sciences, Nagasaki, Japan

## Abstract

Endoscopic submucosal dissection (ESD) permits *en bloc* removal of superficial oesophageal squamous cell carcinoma (ESCC). However, post-procedure stricture is common after ESD for widespread tumours, and multiple endoscopic balloon dilation (EBD) procedures are required. We aimed to evaluate the safety and effectiveness of endoscopic transplantation of tissue-engineered autologous oral mucosal epithelial cell sheets that had been transported by air over a distance of 1200 km in controlling postprocedural oesophageal stricture. Ten patients who underwent complete circular or semicircular ESD for ESCC were transplanted with cell sheets. The safety of the entire process including cell sheet preparation, transport, ESD and cell sheet transplantation was assessed. The incidence of oesophageal stricture, number of EBD sessions, and time until epithelialization were investigated. Each ESD was successfully performed, with subsequent cell sheet engrafting carried out safely. Following cell sheet transplantation, the luminal stenosis rate was 40%, while the median number of EBD sessions was 0. The median post-ESD ulcer healing period was rather short at 36 days. There were no significant complications at any stage of the process. Cell sheet transplantation and preparation at distant sites and transportation by air could be a safe and promising regenerative medicine technology.

## Introduction

Endoscopic submucosal dissection (ESD) allows *en bloc* removal of superficial oesophageal cancer^1^. However, oesophageal stenosis often occurs after ESD when a widespread lesion involves more than three-fourths of the luminal circumference^2,3^. Such situations require frequent endoscopic balloon dilatation (EBD), which compromises quality of life and prolongs hospitalization^1^. The physical dilatation may also carry a risk of perforation. Local steroid (triamcinolone) injection has been effective in avoiding luminal stricture in semicircular but not in complete circular ESD^3^. However, this treatment still carries potential risks of perforation and mediastinal abscess^1,3^. We reported that oral prednisolone could be effective for aggressive oesophageal ESD even in certain cases of circumferential ESD, reducing the number of EBD sessions required^2^. However, prednisolone can cause diverse adverse effects including immunosuppression, diabetes, optical damage, and osteoporosis^1,4^.

Hirose *et al*.^5^ reported a novel cell-harvesting method utilizing poly(N-isopropylacrylamide) (PIPAAm). Various cells adhere, spread, and proliferate on temperature-responsive culture dishes at 37 °C with PIPAAm grafted on. PIPAAm-grafted surfaces are relatively hydrophobic at 37 °C and are highly hydrated below the lower critical solution temperature of 32 °C. In particular, when the temperature is reduced to 20 °C, PIPAAm can swell rapidly, prompting the complete detachment of adherent cells; this enzyme-free harvest process permits the cell sheets to be readily manipulated, transferred, layered, or fabricated^5,6^. With the preserved presence of a deposited extracellular matrix (ECM) on the basal surface of the sheets, they can be transplanted without the need for sutures or clips. Then, the ECM can act as a sticky glue, and the cell sheets can quickly integrate with the transplant sites without sutures in the span of 10 minutes^5–7^. In a canine model, cell sheets can survive and remain attached to oesophageal ulcer wound beds for 8 days^6^.

Thus, Ohki *et al*.^7^ applied endoscopic transplantation of cultured autologous oral mucosal epithelial cell sheets in 9 patients with superficial oesophageal squamous carcinoma to prevent post-ESD stricture. The results were satisfactory as 8 of these 9 patients were free of oesophageal stricture and the procedure was safely performed without complications^7^. Thus, endoscopic cell sheet transplantation can be expected to succeed as a new modality for the prevention of post-ESD stenosis. Nevertheless, this cell-based therapy using autologous oral mucosal epithelial cell sheets also has disadvantages. Fabrication of cell sheets is both technically and financially difficult even in a university tertiary hospital setting^4,7,8^.

One potential solution to these problems is to use ready-made oral mucosal epithelial cell sheets that can be transported from a production site equipped with a cell culture facility (CCF) to a remote hospital that does not have a CCF to fabricate cell sheets, where they will then be transplanted. With this centralized production and distribution system, cell sheet transplantation could be performed in almost all hospitals everywhere in Japan with no need for the hospitals themselves to fabricate the cell sheets in their own CCFs.

**Figure 1:**
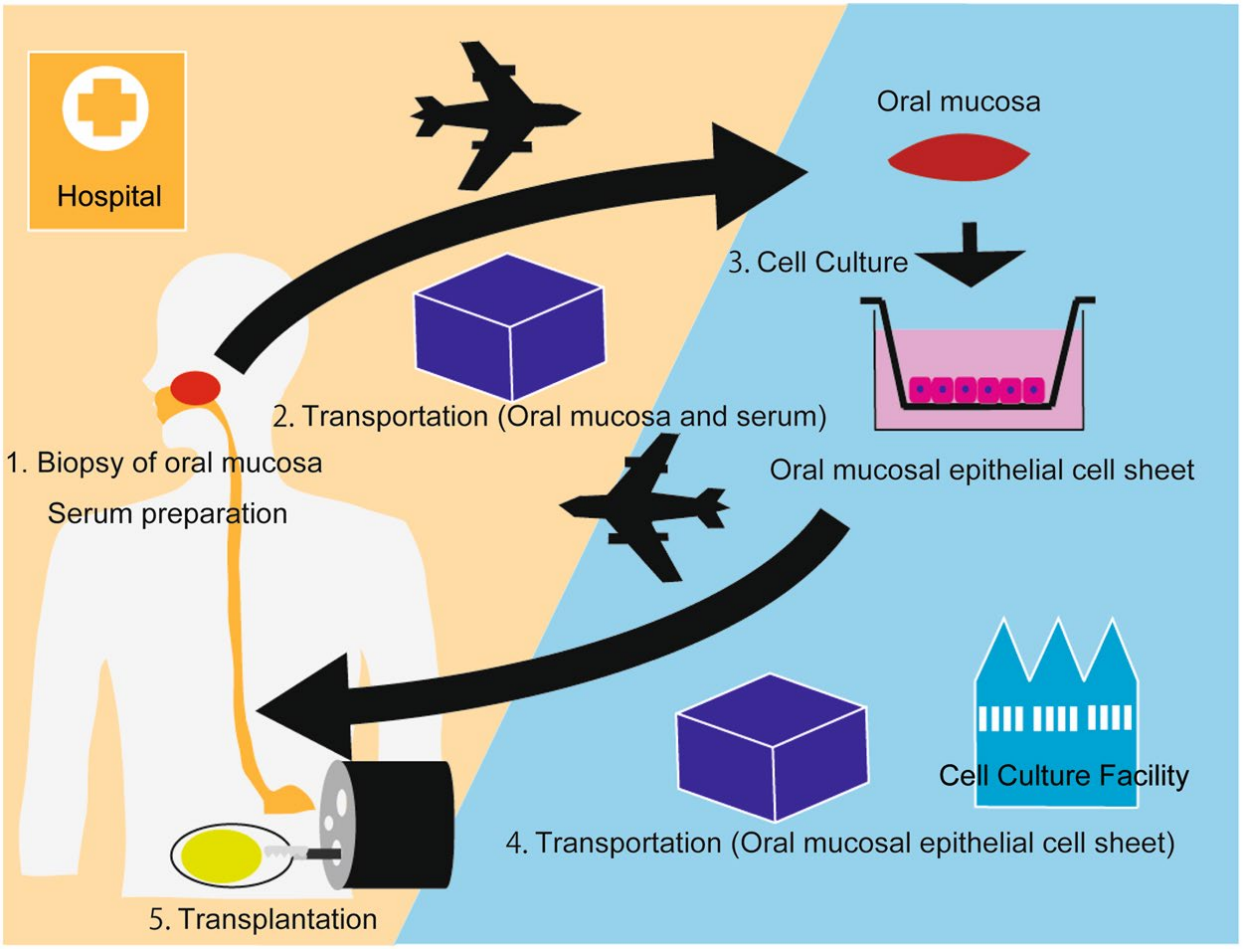
Schematic figure of the fabrication and transplantation of oral epithelial cell sheets and their transportation by air between the hospital and the regenerative institution.

Tissue-engineered oral mucosal epithelial cell sheets resemble the native oesophageal epithelium, with a stratified, squamous, non-keratinised structure and similar expression of cytokeratins^9,10^. In the present study, we therefore aimed to test this possibility by performing oral mucosal epithelial cell sheet transplantation for prevention of post-ESD oesophageal stenosis in co-operation with the Institute of Advanced Biomedical Engineering and Science at Tokyo Women’s Medical University. This study included tissue engineering of transplantable oral mucosal epithelial cell sheets that were fabricated from patients’ own oral mucosa using autologous serum. In addition, the clinical study included transportation of cell sheets between the factory in Tokyo and the hospital in Nagasaki, covering a distance of no less than 1200 km via airplane with a travel time of 7 hours, and suture-less, endoscopic cell sheet transplantation (Fig. 1) on a post-ESD ulcer just after extensive ESD for superficial oesophageal squamous cell carcinoma (ESCC).

## Results

### Preclinical validation of the utility of the newly developed equipment and quality assurance of oral mucosal epithelial cell sheets transported by airplane

In the preclinical validation, the utility of the newly developed equipment and the quality of oral mucosal epithelial cell sheets were verified (Supplemental Figs 1 and 2). There were no adverse events in any of the three healthy volunteer donors during the preclinical validation study. There were no infections inside the containers after transportation. The internal temperature of the second container never dropped below 35 °C in the entire 8 hours, even in the cold environment (Supplemental Fig. 1D). It was important to confirm that the cell sheets were detachable without macroscopic defects or microscopic alterations after airplane transportation. Supplemental Fig. 2F shows the non-disrupted cell sheets with 4–5 cell layers even after transportation. Through a planned clinical study comparing pre- and post-transportation cell sheets made from healthy donor samples, it could be concluded that such air transportation technology is a suitable way to provide a clinical-grade cell sheet safely.

### Air transportation of patient mucosa from Nagasaki to Tokyo and of fabricated cell sheets back to Nagasaki

**Figure 2:**
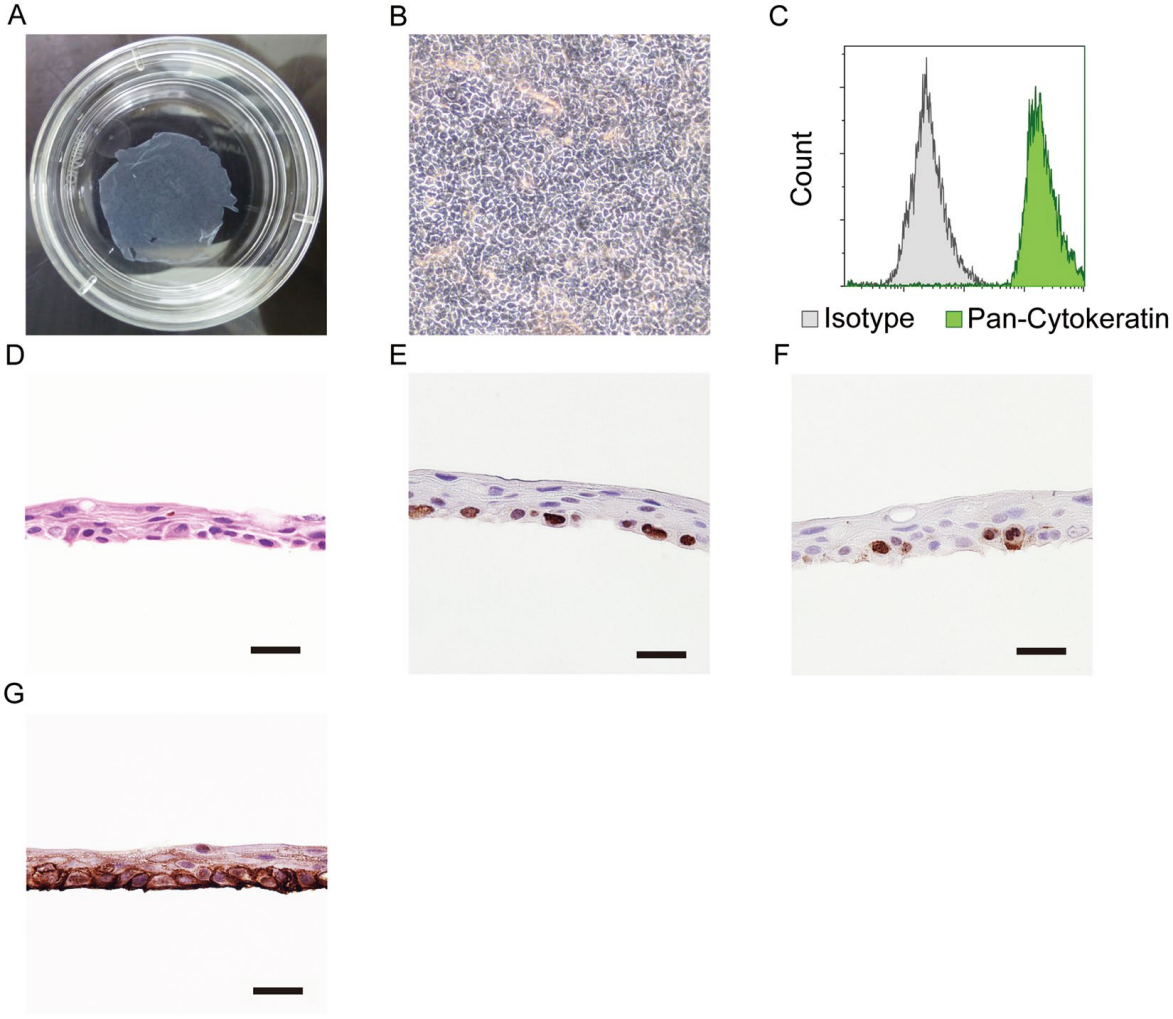
The fabricated oral epithelial cell sheets; there was nominal macroscopic and microscopic alteration (Fig. 2A,B, respectively). The purity of epithelial cells in the fabricated oral epithelial cell sheet was more than 80%. Green shows cytokeratin-positive cells (FITC-conjugated anti-pan-cytokeratin antibody). Grey shows the isotype control (Fig. 2C). Histological examination with haematoxylin and eosin staining showed a layered squamous epithelial cell configuration (Fig. 2D). In the basal layer, there were many sox2-positive progenitor cells (Fig. 2E). Several cells in the basal layer proliferated (Fig. 2F). After being detached from temperature-responsive dish, the oral epithelial cell sheet preserved integrin beta 4, which is an adhesion molecule (Fig. 2G). The scale bars represent 25 µm.

Entire oral mucosal cell sheets for all patients were successfully fabricated at Tokyo Women’s Medical University, reaching confluency on the 8th to the 13th day from the establishment of the culture. All the cell sheets were sterile and free of infection throughout every process, and thus they were transported back to Nagasaki University Hospital. Each cell sheet was detached without no macroscopic defects and microscopic alterations (Fig. 2A,B to be used for case #1). As shown in Table 1, on the day before transplantation, the cell sheets passed all validation criteria, including a total cell count ranging from 2.6 to 9.3 (median, 6.6) × 10^5^ per sheet, viability ranging from 89 to 99% (median, 95%) and purity ranging from 80 to 100% (median, 99%), as measured using flow cytometry with pan-cytokeratin antibody (Fig. 2C). In addition, all the endotoxin levels were below 1.0 EU/mL, and *Mycoplasma pneumoniae* DNA was undetectable from the culture media examined. As a result, there were no significant differences in any of the validation criteria, including the number of cells per sheet, viability and purity, between the stricture and non-stricture cases (Table 1). No complications or adverse events occurred during the process. Histological examination by haematoxylin and eosin staining of representative cell sheet samples showed a layered squamous epithelial cell configuration after transportation (Fig. 2D). Immunohistochemical findings showed that the basal layer of the fabricated oral epithelial cell sheets consisted of sox2-positive cells, contained Ki67-positive cells, and preserved integrin beta 4 (Fig. 2E–G, respectively).

**Table 1:** Cell sheet validation tests and oesophageal stricture after ESD and endoscopic cell sheet transportation and transplantation.

n	Post-ESD stricture	Cell number (×10^5^)	Cell viability (%)	Cell purity (%)	Cell form	Endotoxin levels (EU/mL)
1	No	4.6	99	80	normal	0.028
2	Yes	2.6	95	84	normal	0.209
3	No	7.5	96	96	normal	0.139
4	Yes	8.3	95	100	normal	0.136
5	No	8.3	97	100	normal	0.093
6	No	5.1	89	98	normal	0.031
7	No	3.7	91	99	normal	0.128
8	Yes	7.1	94	99	normal	0.049
9	Yes	9.3	96	100	normal	0.270
10	No	6.0	94	99	normal	0.053

### Oesophageal ESD

The clinicopathological characteristics of the 10 eligible patients with ESCC are summarized in Table 2. Oesophageal ESD was successfully performed *en bloc* without procedure-related bleeding or perforation. The extent, longitudinal diameter and area of resection are listed in Table 2. There were 9 male patients and 1 female patient with a total of 8 middle thoracic and 2 lower thoracic resected areas. The circumference of the resection was more than five-sixths (5/6) of the total circumference in every patient. The mean resected size and area were 63 mm and 3405 mm^2^, respectively. The median and average number of cell sheets transplanted were 6.5 and 7.2, respectively. There was a nominal lag time (less than 2 hours) between the resection and the use of epithelial sheets. The calculation formula, advocated by Ohki *et al*.^4^, was as follows:

$${\rm{Approximate}}\,{\rm{percentage}}\,{\rm{of}}\,{\rm{cell}}\,{\rm{sheet}}\,{\rm{coverage}}\,( \% )\,\fallingdotseq \,\frac{{\rm{area}}\,{\rm{of}}\,{\rm{cell}}\,{\rm{sheets}}\,({\rm{circular}})}{{\rm{area}}\,{\rm{of}}\,\mathrm{ulceration}\,\,({\rm{elliptical}})}=\frac{{(\frac{12}{2})}^{2}x\pi }{1.5\cdot \frac{y}{2}\cdot \frac{z}{2}\pi }\times 100$$

**Table 2:** Outcomes of 10 patients treated by endoscopic submucosal dissection (ESD) and endoscopic transplantation of oral epithelial mucosal cell sheets. Mt, middle thoracic oesophagus; Lt, lower thoracic oesophagus; EP, invasion of epithelium; LPM, invasion through the basement membrane to the lamina propria mucosae; MM, invasion to the muscularis mucosae; and SM2, submucosal invasion (>200 μm into the submucosa).

Cases	Age. Sex	Site of ESD	Circumference of resection	Specimen size (mm)	Resection area (mm^2^)	Number of sheets transplanted	Proportion of cell sheet coverage (%)	Depth of tumour invasion	Post-ESD stricture	Duration of ulcer healing (days)
1	55⋅M	Mt	7/8	80 × 55	4400	6	13.1	LPM	No	28
2	68⋅M	Mt	9/10	75 × 69	5200	7	12.9	LPM	Yes	36
3	73⋅M	Lt	5/6	45 × 30	1350	5	35.6	LPM	No	40
4	58⋅M	Mt	7/8	55 × 46	2530	8	30.4	EP	Yes	29
5	67⋅M	Lt	5/6	50 × 33	1650	8	46.5	LPM	No	29
6	56⋅M	Mt	5/6	55 × 40	2200	6	26.2	LPM	No	29
7	63⋅M	Mt	9/10	73 × 55	4015	8	19.1	MM	No	36
8	72⋅M	Mt	10/10	95 × 84	7985	13	15.6	MM	Yes	172
9	62⋅F	Mt	10/10	53 × 50	2650	5	18.1	SM2	Yes	70
10	74⋅M	Mt	7/8	46 × 45	2070	6	27.8	LPM	No	42

x: number of cell sheets

y: major axis of an elliptical specimen

z: minor axis of an elliptical specimen

The approximate proportion of engrafted cell sheet coverage for the areas removed by ESD ranged from 12.9 to 46.5% (median, 22.7%; mean, 24.5%), as listed in Table 2.

**Figure 3:**
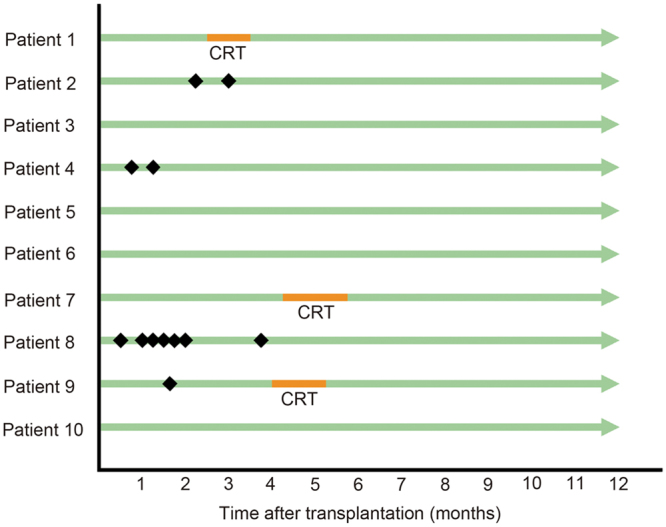
Patients’ outcomes after transplantation of tissue-engineered oral epithelial cell sheets after oesophageal endoscopic submucosal dissection. No patients withdrew from the study. Four patients developed oesophageal stricture and underwent endoscopic balloon dilation (#2, #4, #8, and #9). Refractory luminal stricture, which is defined as more than 5 sessions of balloon dilation, occurred in only one patient (#8) following ESD and cell sheet transplantation. Three patients underwent additive radiotherapy combined with chemotherapy after complete mucosal healing of post-ESD ulceration (#1, #7, and #9). Black squares demonstrate endoscopic balloon dilations. CRT, chemoradiotherapy.

There were no significant differences between the oesophageal stricture and non-stricture cases in clinico-pathological parameters including the resection size and area and the number of sheets transplanted, as summarized in Table 3. Histopathological examination of resected specimens showed curative resection with tumour-free lateral and basal margins in 7 of the 10 patients. Because of a higher risk of nodal metastasis and a probable need for additional therapy, two patients (#1, #7) positive for lymphovascular invasion and one patient (#9) with SM2 invasion of ESCC cells underwent additive radiotherapy totalling 50.4 Gy, leading to histopathologically complete response without complications. Thus, the radiation therapy was conducted for each (#1, #7, and #9) after ESD and cell sheet engraftment (Fig. 3).

**Table 3:** Comparison between stricture and-non-stricture cases after ESD and cell sheet transplantation

	Stricture cases	Non-stricture cases	p-value
Age (mean/median)	65/65 (58~72)	64.7/65 (55~74)	0.9465
Sex (M/F)	3:1	6:0	0.4000
Site of ESD (Mt/Lt)	4:0	3:3	0.0910
Circumference of resection (%) (mean/median)	94.38/95 (87.5~100)	85.82/85.4 (83.3~90)	0.1176
Resection area (mm^2^) (mean/median)	4591.3/3925 (2530~7985)	2614.2/2135 (1350~4400)	0.3055
Number of sheets transplanted (mean/median)	8.25/7.5 (5~13)	6/6 (5~8)	0.4298
Proportion of cell sheet coverage (%) (mean/median)	19.25/16.85 (12.9~30.4)	28.05/27 (13.1~46.5)	0.2287
Depth of tumuor invasion (M/SM)	3:1	6:0	0.1967
Duration of ulcer healing (days) (mean/median)	76.75/53 (29~172)	34/32.5 (28~42)	0.2891

### Clinical outcomes of endoscopic cell sheet transplantation

None of the patients experienced significant clinical signs related to cell sheet transplantation with the exception of transient high-grade fever of more than 38 °C and chest pain for a few days. Thereafter, 6 patients remained free of adverse events up to 67 weeks later and even at the last follow-up in the outpatient clinic (median 105.5, mean 103, weeks; range, 67 to 135 weeks later), whereas 4 patients complained of dysphagia that required a maximum of 7 sessions of EBD with a median of 1.5 sessions (mean 2.75) over the course of 70.5 days (mean 72) (range: 49–98 days) (Fig. 3). Otherwise, no significant complications or adverse events occurred during a median follow-up period of 105.5 (mean 107) weeks. Additionally, no abnormal laboratory data were observed in relation to cell sheet transplantation with the exception of acute inflammatory reactions, manifested by parameters including C-reactive protein (CRP) levels, circulating white blood cell (WBC) counts, and neutrophil counts. The peak CRP level had a median of 4.1 (mg/dL, average 5.7), ranging from 1.95 (patient #6) to 13.95 (#8); the peak WBC count had a median of 8850/mm^3^ (average 9680), ranging from 7600 (#4) to 13900 (#9); and the peak neutrophil count had a median of 6880/mm^3^ (average 7570), ranging from 5780 (#4) to 11260 (#9).

There was no reoccurrence of disease in any of the patients after treatment with the cell sheets. We have not identified any tumours, including locally recurrent squamous cell neoplasm or teratoma, during the follow-up period. Annual systemic computed tomography (CT) scans were performed to detect nodal and distant metastases, and there were no metastases in any of the 10 patients during the follow-up period.

Furthermore, the median ulcer healing period until epithelialization was 36 days (mean 51.1), ranging from 28 to 172 days. Compared with the 6 patients without post-ESD stricture and related dysphagia, the 4 patients suffering from luminal stricture did not have a significantly different ulcer healing period (Tables 2 and 3).

**Figure 4:**
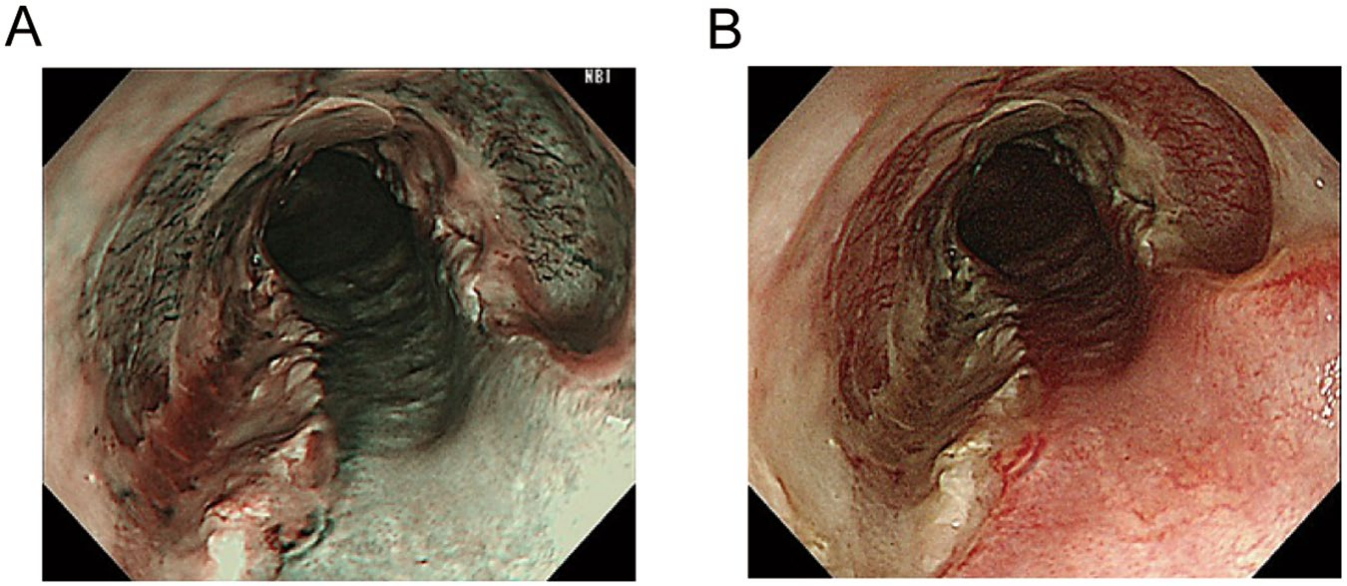
On the 4^th^ day after transplantation, narrow-band imaging endoscopy enabled distinct identification of the transplants, which were accompanied by plenty of vascularization at the transplantation sites (Fig. 4A), in images corresponding to the white light-guided endoscopic images (Fig. 4B).

All the engrafted cell sheets were present on the 4^th^ day after transplantation in the 8 cases examined. NBI endoscopy enabled distinct identification of the transplants accompanied by plenty of vascularity at the transplantation sites (Fig. 4). We did not perform endoscopy in 2 of the patients on the 4th day after ESD, but these 2 patients, similar to the remaining 8 patients, underwent a periodic endoscopic follow-up to confirm the presence of cell sheets on the 7th day (and 2, 4, 12, 24, and 48 weeks later) following ESD and cell sheet engraftment. Thus, all 10 patients underwent NBI-based endoscopy on the 7th day after ESD and engraftment. The cell sheets were still present at the 1-week but not the 2-week follow-up endoscopy with NBI in each case.

Upon follow-up endoscopy, the post-ESD sites in all 4 stricture cases were still covered with white exudate even 4 weeks after transplantation. On the other hand, in 5 of the 6 non-stricture cases, repeated endoscopy did not identify any such white exudate on the post-ESD sites after 2 weeks. According to the definition proposed by Kockman *et al*.^11^, one patient (#8) experienced refractory luminal stricture after ESD and cell sheet transplantation; the patient required 7 sessions of EBD to remediate the luminal stricture.

**Figure 5:**
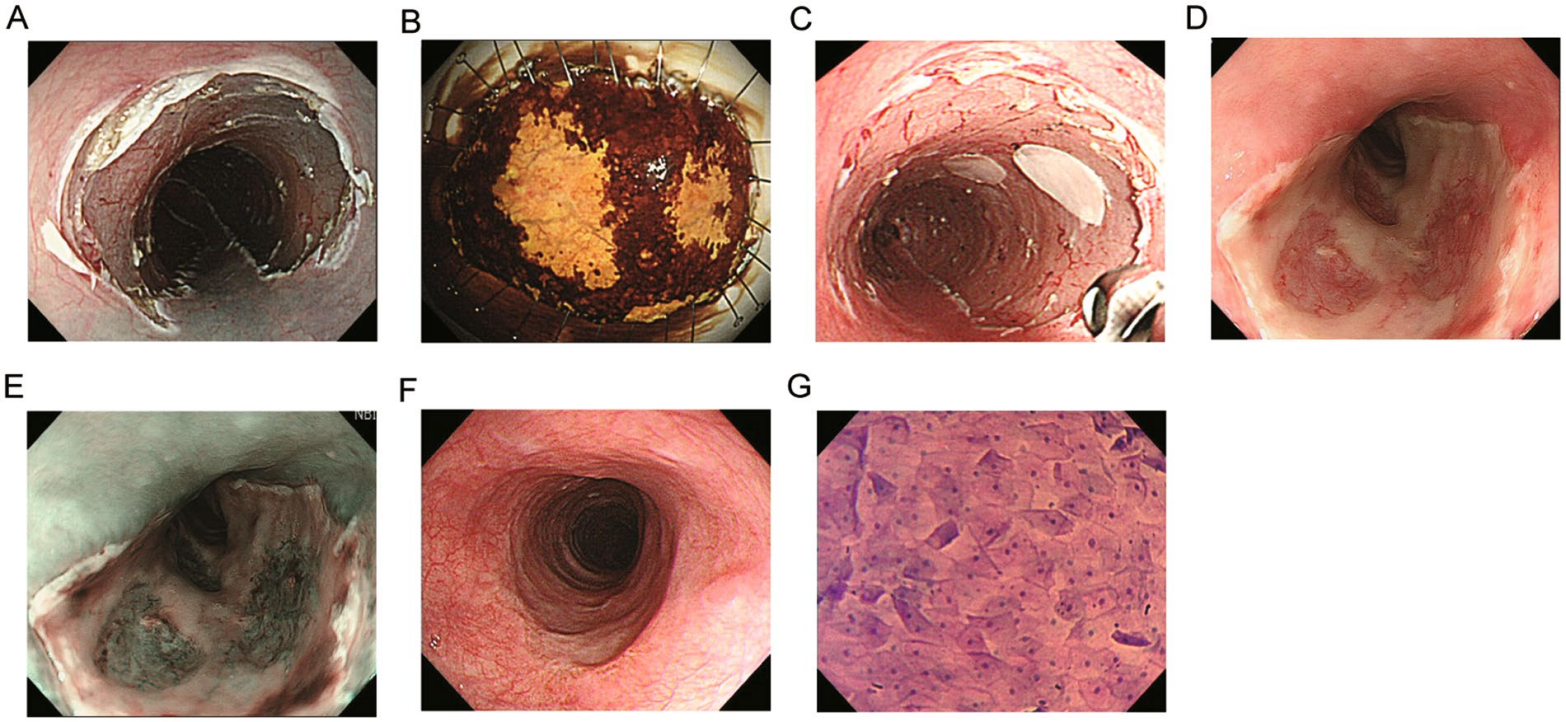
In case #1, semicircular endoscopic submucosal dissection (ESD) spanning seven-eighths of the luminal circumference was successfully performed for middle thoracic oesophageal squamous cell carcinoma (ESCC) (Fig. 5A), and the two ESCC lesions within invasion depth of the lamina propria were both detected by iodine staining (Fig. 5B) in the removed specimen. Six cell sheets were endoscopically transplanted (Fig. 5C). On the 4^th^ day after transplantation, the engrafted cell sheets were present on the post-ESD site (Fig. 5D). NBI-equipped endoscopy enabled distinct identification of the transplants accompanied by plenty of vascularity at the transplantation site with no white exudate (Fig. 5E). Twenty-eight days later, complete ulcer healing with epithelialization was achieved without luminal stricture (Fig. 5F). The transplanted sites were densely covered with regular-shaped squamous cells when observed using endocytoscopy (Fig. 5G). The nuclei of the cells showed nominal abnormality in size and configuration.

In the 5 patients without oesophageal stricture, the endocytoscopic findings at approximately 400-fold magnification were assessed using the ECA classification^12^, which ranges from ECA1, with a normal nuclear/cytoplasmic (N/C) ratio and regular cell arrangement, to ECA5, with an abnormal N/C ratio and irregular cell arrangement corresponding to ESCC. There were 2 cases of ECA1 (Fig. 5) and 3 of ECA2.

### Changes in vascular endothelial growth factor (VEGF) levels in plasma and supernatant culture medium

We did not obtain any biopsies from the engrafting sites of the cell sheets to measure cytokines and growth factors. Instead, the plasma VEGF levels were measured the day before ESD and cell sheet engraftment (Day 0), one day later (Day 1), and on Days 3, 7, and 28. There was significant difference in plasma VEGF levels between Day 1 and Day 3 after ESD and cell sheet engraftment (Supplemental Fig. 3A). As the oral mucosal cell sheet fabrication was performed using autologous sera, we also confirmed the significant elevation of VEGF levels in the supernatant culture medium during cultivation, from the initial day of cell sheet fabrication (called ‘Medium Day 0’) to the 15^th^ day of cultivation (called ‘Medium Day 15’); those media were also compared with the serum obtained 23 days prior to the cell sheet engraftment (called ‘Serum’ in Supplemental Fig. 3B). There were significant differences in VEGF levels between ‘Serum’ and ‘Medium Day 15’ and between ‘Medium Day 0’ and ‘Medium Day 15’. Among the various pro-inflammatory cytokines, chemokines and growth factors assessed, interleukin (IL)-12 (p70) and VEGF levels were significantly elevated in the culture supernatant.

### Case Presentations

In case #1 (Table 1), semicircular ESD with 7/8 circumference was successfully performed for middle thoracic ESCC (Fig. 5A,B), and 6 cell sheets were endoscopically transplanted (Fig. 5C). On the 4^th^ day after transplantation, the engrafted cell sheets were present on the post-ESD site (Fig. 5D). Narrow-band imaging (NBI) endoscopy enabled distinct identification of the cell sheets accompanied by much vascularity at the corresponding transplantation sites (Fig. 5E). Twenty-eight days later, complete ulcer healing with epithelialization was achieved without luminal stricture (Fig. 5F). The transplantation sites were densely covered with regular-shaped squamous cells when observed using endocytoscopy (Fig. 5G). The nuclei of the cells showed nominal abnormality in size and configuration. The 48-week clinical course and the last outpatient clinic visit (29 months later) were uneventful without related dysphagia. (Fig. 5, Supplemental Fig. 4, Supplemental Fig. 5).

## Discussion

In all cases within the current study, we succeeded in transporting cell-based tissue engineering products by airplane and successfully used those products for cell transplantation. By follow-up endoscopic and histological examinations, we also demonstrated rapid epithelization and reconstruction of oesophageal mucosa following transplantation. It was proposed that refractory stricture be defined as follows: an anatomic restriction because of cicatricial luminal compromise or fibrosis that results in the clinical symptom of dysphagia. This may occur as the result of an inability to successfully remediate the anatomic problem to a diameter of 14 mm over 5 sessions at 2-week intervals^11^. On the basis of this definition, refractory luminal stricture occurred in one patient (10%, 1/10) following ESD and cell sheet transplantation. This patient (#8) underwent fully circumferential ESD and had the largest resection in both length and area, which may be why they required such frequent EBD sessions.

We compared the outcomes of the present study with those of the other standard studies that have documented the incidence of post-ESD stricture along with the extent of resection performed in test subjects. Currently, preventive strategies for post-ESD stricture include EBD, triamcinolone local injection, oral prednisolone administration and stent insertion^1,13^. Ono *et al*.^14^ reported that among 107 superficial oesophageal squamous cell neoplasms in 84 patients treated by ESD, 15 (18%) patients experienced oesophageal stricture. However, the cohort included only 10 (9.3%) cases whose ESD extended more than 3/4 of the circumference of the lumen, and when analysis was limited to that subset, 9 (90%) patients experienced oesophageal stricture requiring periodic EBD. Notably, one patient with a nearly complete circular resection underwent 20 sessions of balloon dilation. By contrast, all the cases in our study had more than five-sixths of the luminal circumference resected using oesophageal ESD. In another study that we conducted, 43 patients with superficially extended ESCC who underwent semicircular (more than three-fourths but not the full circumference) or complete circular ESD were enrolled, and the primary results showed that the number of additional EBD sessions after the 8-week planned preemptive EBD was significantly higher in the preemptive EBD group than in the oral prednisolone group^2^. In the oral prednisolone group, 1 out of 19 (5.3%) patients experienced post-ESD stricture. However, after accumulating the cases in our hospital, we found that 13 out of 84 (15.5%) and 9 out of 61 (14.8%) experienced luminal stricture in the oral prednisolone and triamcinolone local injection groups, respectively^15^. Among patients with wholly circumferential ESD, 7 out of 24 (29.2%) and 3 out of 3 (100%) experienced an oesophageal stricture for each group^15^. Again, the average number of EBD sessions required was 15.6 in the preemptive EBD group and 1.7 in the oral prednisolone group^2^. In this cell sheet study, a mean of 2.75 sessions of EBD were required. Notably, the ulcer healing period until re-epithelialization was 36, 61 and 66 days for cell sheet engraftment, triamcinolone and oral prednisolone, respectively^15^. Considering these results, we cannot conclude solidly that cell sheet therapy can offer a safer treatment option to prevent oesophageal strictures after extensive ESD by allowing faster healing until epithelialization, despite the preliminary efficacy. Full verification of efficacy will require a prospective comparative trial with the appropriate controls. It was reported that biodegradable stents composed of poly-L-lactic acid prevented oesophageal strictures after ESD in 2 patients^16^. However, it may be insufficient to cover an extensive mucosal defect after oesophageal ESD. Currently, there is insufficient clinical evidence on the use of stent insertion to prevent oesophageal strictures, particularly following extensive ESD. Accordingly, one may suppose that the stent strategy should not be considered standard^1^. Recently, the use of amniotic membranes^17^, polyglycolic acid sheets^18^, or a decellularized tubular scaffold^19^ was also reported for the placement of oesophageal mucosa in preclinical and small clinical studies.

Of clinical significance, we confirmed the safety of autologous mucosal cell sheet transplantation as shown in a prior human Phase I study^7^. Repeated physical and laboratory examinations during the 48-week follow-up showed nominal adverse effects through every part of the process, which consisted of cell sheet fabrication, transportation and transplantation. Notably, this study included air transportation of the patient mucosa from Nagasaki to Tokyo and return air transportation of the fabricated cell sheet from Tokyo to Nagasaki (Fig. 1). Hence, the autologous oral mucosal cell sheets could be transplanted even following transportation over a distance of no less than 1200 km between the two institutes. The travel time on the route was less than 7 hours in this study. In fact, the quality of the tissue-engineered cell sheets with regard to viable cell numbers and purity of squamous epithelial cells at the destination, on the day of ESD, immediately prior to cell sheet transplantation, remained quite high and was equal to the quality of the sheets at Tokyo Women’s Medical University the day before they were transported. Following airplane transportation, the cell sheets preserved the macroscopic appearance of sheet formation and configuration, retained microscopic structures consisting of layered squamous epithelial cells, and passed validation tests including the number of cells (10^5^ or more cells per sheet), viability (<70% negative for trypan blue) and purity (>70% positive for FITC-conjugated anti-pan-cytokeratin antibody). Furthermore, the cell sheets were successfully fabricated without any identifiable infections, and nominal problems occurred throughout each process^4,8^.

Repeat oesophagoscopy showed that the fabricated cell sheets were endoscopically transplanted with no carriers or sutures and were still extant beyond 4 days after transplantation, until at least 1 week later. Interestingly, the engrafting locations of the cell sheets appeared distinct and more easily identifiable under NBI-enhancement endoscopy (Figs 4 and 5 and Supplemental Fig. 4), possibly reflecting copious vascularity. The copious vascularity may be associated with VEGF production, as substantial alterations were observed not only in the culture medium but also in circulation (Supplemental Fig. 3). This may be linked to facilitation of healing, considering previous studies^20^, and should be a topic for further research in future studies. The ulcer healing period lasted for a median of 36 days before epithelialization. Although oral prednisolone intake showed comparable effects for stricture prevention after oesophageal ESD, the ulcer healing period was rather long at over 2 months (61 days; range, 31–245 days) in a historical comparison study at the same institute^15^. Ohki *et al*.^7^ also reported that epithelialization started at 1 week following oesophageal ESD and cell sheet transplantation on the day of ESD, and subsequently the postprocedural ulcer was healed after 3.5 weeks. Histological examination demonstrated regenerated oesophageal tissue covered by squamous cell epithelia^7^. As presented in the current study, the regenerative mucosa maintained stemness and proliferation upon immunohistochemical analyses for sox2 and ki67, respectively^21,22^. In a human transplantation study of tissue-engineered cell sheets for stricture prevention after ESD for patients with Barrett’s-oesophagus-associated neoplasia, Jonas *et al*.^23^ have also reported that the harvested oral mucosal epithelial cell sheets consisted of cytokeratin-positive cells with high average purity of 94.55 ± 4.45%. According to their study, laminin was also more localized to the basal parts of the sheets, consistent with a normal epithelium. The gap junction protein connexin 43 was also localized to the basal part of the cell sheet. Ki-67-positive proliferating cells were mostly localized to the basal part of the sheet as in normal epithelium. Transmission electron microscopy revealed a multilayered cell configuration with several desmosomes. Nevertheless, there might be the possibility of initially undetected teratoma, requiring careful longer-term follow-up, as oral epithelial cell sheets express pluripotency markers including oct-4 and sox-2^23^.

Again, regular-shaped squamous cells without abnormality in their N/C ratio had grown over the transplanted sites when observed using endocytoscopy^12^. In 3 cases, the regenerative oesophageal lumens also remained in good condition after radiation therapy combined with chemotherapy. Thus, sheets of tissue-engineered epithelial cells fabricated *ex vivo* from autologous oral mucosa seem quite effective for reconstructing the oesophageal surface by facilitating ulcer healing. In addition, endocytoscopy can be a useful modality for observation of epithelialization following cell sheet engraftment without mucosal damage due to biopsy sampling.

Factors associated with oesophageal stricture despite cell sheet transplantation remain to be determined owing to the study limitations. This study was conducted to investigate the clinical safety (the main outcome measurement) and the efficacy of cell sheet transplantation after airplane transportation of the fabricated cell sheet. There were no significant differences in background parameters including cellular qualities and clinical characteristics between the small case series of stricture and non-stricture patients. Wound environments such as excessive inflammation, bile reflux, and infections in the acute phase might also affect stricture formation^6^. Obviously, complete circular ESD still seems recalcitrant and uncontrollable even with long-term, systemic administration of steroids^1,13^. In this regard, we calculated the percentages of engrafted cell sheet coverage for the areas removed by ESD as having a mean value of no more than 24.5%, as mentioned above. By contrast, the average cell sheet coverage in the 9 patients reported by Ohki *et al*.^4,7^ was substantially higher, with no fewer than 39.3% of the patients reaching 83.1% coverage. More recently, Jonas *et al*.^23^ transplanted oral mucosal cell sheets for widespread lesions of Barrett’s-oesophagus-associated premalignant lesions and adenocarcinoma at the oesophago-gastric junction within the same institute containing a CCF, but among 5 patients who received cell sheet transplantation, luminal stricture occurred in all 3 of the patients in whom wholly circumferential ESD was performed. If greater numbers or areas of cell sheets could be fabricated and transplanted, this might result in less frequent occurrence of luminal stricture and might therefore lead to milder and less frequent related symptoms. Improvements and innovations in cell sheet technologies to resolve this issue and to decrease the occurrence of luminal stricture following cell transplantation are currently under way.

It was also beyond the scope of the present study to investigate whether systemic steroid administration (oral prednisolone) or tissue-engineered cell sheet transplantation might be the more effective treatment option in controlling oesophageal stricture following extensive ESD, in particular circumferential ESD. Further multicenter prospective studies with larger case series are warranted for this purpose. These types of studies are quite expensive because of the strict standards of GMP; hence, facilities equipped with CCFs for cell sheet fabrication are still limited^4,8^. The latter problem was the other reason for the current study, despite the fact that a great deal of labour was required for its performance. Nevertheless, cell sheet transplantation technology has now been applied to other systems including autologous corneal reconstruction using keratinocytes for bilateral ocular trauma^24^. Encouraging studies are also being reported in the progression of the recovery of heart functions following transplantation of autologous myoblast sheets^25^. More recently, adipose-tissue-derived stromal cell sheet transplantation was attempted for oesophageal stricture prevention after ESD in a porcine model^19^. Allogeneic transplantations of tissue-engineered skin products are widely used for skin burn treatment in humans, and these cell sheets are another candidate source of cells for transplants as they have shown efficacy for stricture prevention following ESD in a swine model in the same setting as the present study^26^. This would be a future topic of interest for standardizing treatment using epithelial cell sheets. Cell-based therapy using autologous oral mucosal epithelial cell sheets has several disadvantages compared with anti-inflammatory drug therapy using steroids. Indeed, it is idealistic to suppose that the ready-made oral mucosal epithelial cell sheets can consistently be transported in a suitable environment from a production site to the hospital where the transplantation will be performed. However, the operating cost for CCF is still high and is currently estimated to be at least 20000 to 30000 USD per case^8^. Thus, fabricating oral mucosal epithelial cell sheets in a CCF in every hospital would be technically and financially difficult.

In conclusion, cell sheets can be fabricated at a distant site and safely transported to a site that is not equipped with a CCF. It has not yet been conclusively determined whether cell sheet transplantation is more effective than steroid administration in treating oesophageal stenosis; larger prospective studies are required and are currently being undertaken in multicenter controlled settings to address this question.

## Methods

### Patients

Ten ESCC patients who underwent ESD with more than three-fourths of the circumference resected and who required treatment for oesophageal stenosis underwent cell sheet transplantation. Cell sheet transplantation was performed from July 2013 to October 2014. This study was approved by the Ministry of Health, Labour and Welfare of Japan on March 29^th^ (#0329-21), 2013 and was conducted until October 31^st^, 2015. Since cell sheet grafts may include squamous epithelial stem cells, this study was conducted in accordance with the guidelines on clinical research using human stem cells as set by the Ministry of Health, Labour and Welfare. This study was approved by the ethics committees of Nagasaki University (approval number 12090375) and of Tokyo Women’s Medical University (approval number 121006). This study was conducted as a clinical trial registered at the University Hospital Medical Information Network Center (clinical trial registration No. UMIN000010251; receipt No. R000011998); hence, a detailed protocol as of the date of registration (1/April/2013) is shown in a supplemental file, Clinical Research Protocol. Participants were required to fulfil all of the inclusion criteria at enrolment. Patients who met any of the exclusion criteria were ineligible.

As shown in the supplemental file, Clinical Research Protocol, autologous blood collection to obtain serum was performed as follows. We determined the amount of blood to be taken, considering the anticipated number of cell sheets required, and we confirmed that the patient met the requirements regarding general medical conditions and haemoglobin levels. After making these determinations, we collected the blood samples from the patients. We transported the obtained serum to the Institute of Advanced Biomedical Engineering and Science, Tokyo Women’s Medical University.

We harvested oral mucosal tissue from each patient approximately 16 days before the scheduled surgery date as follows. After local anaesthetic injection, oral mucosal tissue was obtained with a surgical knife or by punch biopsy punch according to the number of sheets required, taking into account the ulcer area estimated from the ESD resection margin (approximately 20 mm^2^/sheet, 4 - 6 sheets). We performed compression haemostasis and sutured the injured site. The harvested tissue was placed in PBS, and then the container was placed on ice and transferred into a biological safety cabinet. We disinfected the tissue with iodine and washed it twice. After immersion of the washed and disinfected tissue, it was tightly sealed in its container, kept at approximately 4 °C using cold compresses, and then transported to the CCF of Tokyo Women’s Medical University. Written informed consent was obtained from all 10 subjects before enrolment.

### ESD

Oesophageal ESD procedures were conducted using the same endoscopic and electronic devices previously described and using similar medication protocols and surgical conditions^2^. Each patient received sodium rabeprazole at a standard dose following ESD at least until ulcer healing with epithelialization or relief of the related symptoms.

The depth of tumour invasion, assessed by histopathological examination of the resected specimen, was defined as follows: EP, invasion of the epithelium; LPM, invasion through the basement membrane to the lamina propria mucosae; MM, invasion to the muscularis mucosae; SM1, submucosal invasion (≤200 μm below the muscularis mucosae into the submucosa); and SM2, submucosal invasion (>200 μm into the submucosa).

### Tissue-engineered cell sheet fabrication and endoscopic transplantation

In accordance with a previous human clinical study, autologous oral mucosal epithelial cell sheets were fabricated using the patients’ own serum and oral mucosa at Tokyo Women’s Medical University under the administrational standard operating procedures. The CCF has achieved the standards of good manufacturing practice (GMP)^4,8^. Moreover, a standard operating procedure (SOP) was prepared to optimize the working environment in the CPF and the preparation of the cell sheets^27,28^. First, 23 days before transplantation, autologous serum was collected to be used instead of foetal bovine serum. 3T3 feeder cells derived from murine fibroblasts were not used in the current study^4,8^. Next, 16 days before transplantation, we collected oral buccal mucosa by a surgical procedure performed by a professional dentist (IA) at Nagasaki University Hospital. As mentioned previously, the patient’s oral mucosa was surgically collected was transported over a distance of 1200 km to Tokyo Women’s Medical University on the same day via airplane. As described previously, within the institutional CCF, oral mucosal epithelial cells were enzymatically isolated from the mucosal specimen and suspended as single cells. The cells were then seeded on temperature-responsive cell culture inserts (CellSeed, Tokyo, Japan) that were grafted with PIPAAm. Suspended cells were cultured with modified keratinocyte-conditioned medium for 15 days at 37 °C in a humidified atmosphere containing 5% CO_2_. At 37 °C, the patients’ own isolated, confluent epithelial cells were attached to and grown on the hydrophobic PIPAAm plate until cell sheet formation. Cells were inspected visually under a phase-contrast microscope at each medium change. On Day 2 before transplantation, the cell sheets were required to be detachable. In addition, they were required to preserve the macroscopic appearance of the sheet formation and configuration and retain microscopic structures consisting of layered squamous epithelial cells. They were also required to pass all validation tests including the number of cells (10^5^ or more cells per sheet), viability (<70% negative for trypan blue) and purity (>70% positive for FITC-conjugated anti-pan-cytokeratin antibody (Ks pan 1–8, Progen, Heidelberg, Germany) using flow cytometry (Gallios; Beckman Coulter, Palo Alto, Calif., USA). Sterility was defined as having an endotoxin level below 1.0 EU/mL and testing negative for *Mycoplasma pneumoniae* DNA in the discarded culture medium^4,7,8^. The tissue-engineered cell sheets were then transported back from the CCF in Tokyo Women’s Medical University to Nagasaki University Hospital the day before transplantation.

The ready-to-be-transplanted cell sheets were transported using originally developed equipment consisting of three containers as shown in Supplemental Fig. 1 and harvested by reducing the temperature to 20 °C while oesophageal ESD was ongoing, after which they were transplanted onto the post-ESD ulcer site immediately after ESD. The endoscopic suture-less transplantation protocol was performed in accordance with a previous human clinical study^7^.

For EBD, we used a CRE balloon dilator (Boston Scientific, Boston, Mass); in cases of distinct dysphagia, EBD was performed until the symptom was resolved^2^.

### Follow-up examinations

Follow-up examinations included scheduled repeated endoscopy and clinical symptoms and laboratory data monitoring. Repeated endoscopy using the GIF-Q260J gastrointestinal videoscope with a 9.9 mm diameter (Olympus, Tokyo) was scheduled on Day 4 after transplantation in 8 of 10 patients and at 1, 2, 4, 12, 24 and 48 weeks after the day of ESD and cell sheet transplantation or at any time when dysphagia occurred, as well as at study cessation, to assess the ulcer healing process and the condition of the passage for upper endoscopy in all of the patients. Narrow-band imaging (NBI)-assisted endoscopy was performed to enhance microvascular architectures, and endoscopic ultrasound was also performed for lesions with the probability of submucosal invasion; all patients with lesions involving the muscularis mucosa were included in this study analysis. Vascular architecture was also observed in the healing process using NBI-equipped endoscopy. In 5 of the 6 cases without luminal stenosis, ultramagnification endoscopic observation using endocytoscopy with an integrated prototype (GIF-Y0001; Olympus) followed by 0.05% crystal violet and 1% methylene blue staining was conducted by one experienced endoscopist (HI) after complete ulcer healing. We did not obtain any biopsies from the cell sheets after engraftment, as it was possible that endoscopic biopsy sampling might cause mucosal damage during the healing process. Clinical symptoms including dysphagia, oesophageal pain, oesophagitis and oesophageal infection, as well as vital signs, were checked periodically in the corresponding period. Periodic laboratory data included haematological and biochemistry tests, including renal and liver functions at 1, 2, 4, 12, 24 and 48 weeks after transplantation. We assessed acute inflammatory reactions including circulating CRP, WBC counts, and neutrophil counts as part of the follow-up laboratory data. Chest X-rays were taken on the same schedule. Contrast-enhanced CT images of the neck, chest, and abdomen were examined annually to detect nodal and distant metastases.

### Main outcome measurement

This study was conducted as a non-randomized, single-arm, prospective clinical trial at a university hospital. The primary endpoint was clinical safety in every process including oral surgical sampling of the patients’ own mucosa for cell sheet fabrication, oesophageal ESD, endoscopic transplantation of the tissue-engineered autologous mucosal epithelial cell sheets and follow-up examinations over 48 weeks, as we assessed any complications and the related adverse events. We investigated the efficacy of the treatment in preventing post-ESD stenosis by measurement of the following: the incidence of oesophageal stricture after ESD, the number of EBD sessions required to relieve dysphagia and the ulcer healing period until epithelialization.

Second, refractory luminal stricture was defined as previously described^11^.

### Histology and immunohistochemistry

Paraffin-embedded sections of the fabricated cell sheets and biopsies were stained by haematoxylin and eosin. The protein expression of specimens was evaluated by immunohistochemistry. After the pretreatments, each section of the biopsies was stained with a mouse monoclonal antibody against Sox2, also known as sex determining region Y-box 2, a stem cell pluripotency transcription factor^21,29^ (20G5, Abcam, Cambridge, UK); a rabbit monoclonal antibody against Ki67, which is known as a proliferation marker (SP7, Abcam); and a rabbit polyclonal antibody against integrin beta 4, which is known as a cell adhesion molecule (4707, Cell Signaling Technology, Beverly, MA, USA). HRP-conjugated secondary antibodies were used (ChemMate ENVISION kit, Dako, Glostrup, Denmark) and detected according to the manufacturer’s protocol. The stained sections were observed by microscopy (x400 and x200 magnification, DP70, Olympus).

### Assay of cytokine and growth factor concentrations

Detection and quantification of cytokines and growth factors in samples were performed using the Bio-Plex Human Cytokine GI 27-plex assay (Bio-Rad Co., Hercules, CA). Targets included IL-1β, IL-1ra, IL-2, IL-4, IL-5, IL-6, IL-7, IL-8, IL-9, IL-10, IL-12 (p70), IL-13, IL-15, IL-17, basic fibroblast growth factor, eotaxin, granulocyte colony-stimulating factor, granulocyte–macrophage colony-stimulating factor, interferon-γ, interferon γ-induced protein 10, monocyte chemotactic protein-1, macrophage inflammatory proteins 1α and 1β, platelet-derived growth factor-BB, regulated on activation normal T cell expressed and secreted (RANTES, also known as CCL5), tumour necrosis factor-α and VEGF. Experiments were conducted in accordance with the manufacturer’s instructions. First, 50 μl of beads were added to each well in 96-well microplates. After one wash, 50 μl of standard or sample was added to each well and incubated for 1 hour with shaking. After a wash with wash buffer, 25 μl of biotin-labelled detection antibody was added, and the plates were incubated for 30 min with shaking. After a wash, 50 μl of streptavidin-PE was added, and the plates were incubated for 10 min with shaking. Finally, the beads in each well were resuspended in 125 μl of assay buffer, and the entire plate was read on a Bio-Plex system.

### Statistical analysis

The statistical significance of comparisons between the groups was assessed by Fisher’s exact test in Table 3 (age, sex, site of ESD, depth of tumour invasion). Student’s t-test was used for Supplemental Fig. 2D; a paired t-test was used for Supplemental Fig. 3A,B, and the Mann-Whitney test was used for circumference of resection, resection area, number of sheets transplanted, proportion of cell sheet coverage, and duration of ulcer healing in Table 3. *P* values less than or equal to 0.05 were considered significant.

## Electronic supplementary material

supplemental information
