# Supplementary material for: Oral epithelial cell sheets engraftment for esophageal strictures after endoscopic submucosal dissection of squamous cell carcinoma and airplane transportation
Source: Sci Rep. 2017 Dec 12;7:17460. doi: 10.1038/s41598-017-17663-w (PMC5727129; doi:10.1038/s41598-017-17663-w)
Supplement: Supplementary file 1 — supplemental information [file 41598_2017_17663_MOESM1_ESM.pdf]

Supplemental file information

Oral epithelial cell sheets engraftment for esophageal strictures after endoscopic submucosal dissection of squamous cell carcinoma and airplane transportation

Naoyuki Yamaguchi[1]*, Hajime Isomoto[2]*, Shinichiro Kobayashi[3]*, Nobuo Kanai[4], Kengo Kanetaka[3], Yusuke Sakai[3], Yoshiyuki Kasai[4], Ryo Takagi[4], Takeshi Ohki[5], Hiroko Fukuda[1], Tsutomu Kanda[2], Kazuhiro Nagai[6], Izumi Asahina[7], Kazuhiko Nakao[1], Masayuki Yamato[4], Teruo Okano[4], and Susumu Eguchi[3]

[1]Department of Gastroenterology and Hepatology, Nagasaki University Hospital, Nagasaki, Japan; [2]Division of Medicine and Clinical Science, Department of Multidisciplinary Internal Medicine, Tottori University Faculty of Medicine, Yonago, Japan; [3]Department of Surgery, Nagasaki University Graduate School of Biomedical Sciences, Nagasaki, Japan; [4]Institute of Advanced Biomedical Engineering and Science, Tokyo Women's Medical University, Tokyo, Japan, [5]Department of Surgery, Institute of Gastroenterology, Tokyo Women's Medical University, Tokyo, Japan, [6]Transfusion and Cell Therapy Unit, Nagasaki University Hospital, Nagasaki, Japan; [7]Department of Regenerative Oral Surgery, Nagasaki University Graduate School of Biomedical Sciences, Nagasaki, Japan

Supplemental Figure 1: The ready-to-use cell sheets fabricated in the CPF were transferred to primary containers filled with keratinocyte culture medium containing 5% autologous human serum, where they adhered tightly to the temperature-responsive cell culture inserts. The primary containers were capped tightly to avoid microbial contamination during transportation and were transferred into the secondary container, which consisted of a metallic cage (A). This container had a warmer consisting of a power switch, a lithium-ion rechargeable battery (battery capacity: 5 Ah [output currents of up to 20 A], voltage: DC 10 V-12.6 V), a metal plate (150 mm square), and a thermal regulator (B). The secondary container packed with the temperature-responsive cell culture inserts was placed in the tertiary container (C). As a test of temperature stability, the secondary container had its warmer set to 37 °C and was then placed in the third container and left in the cold room at approximately 4 °C. The internal temperature of the secondary container never fell below 35 °C during the entire 8-hour test, despite the cold environment (D).

Supplemental Figure 1

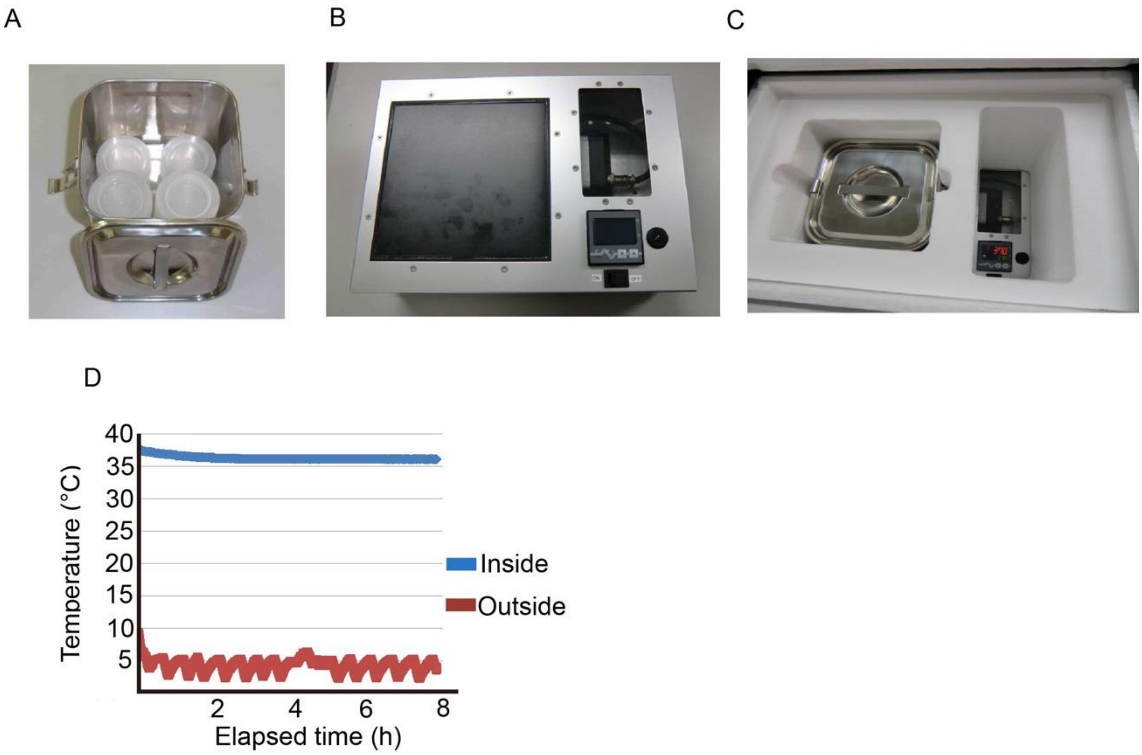

Supplemental Figure 2: We evaluate the safety and quality, both before and after transportation, of oral mucosal epithelial cell sheets fabricated from healthy volunteer donors. Oral and written informed consent was obtained from three healthy volunteers. The preparation of an oral mucosal epithelial cell sheet in the CCF according to SOP was conducted in the same way described in the methodology. Observation under a microscope showed that the epithelial cells in the cell sheet retained normal morphology after transportation (A). After incubation at room temperature, epithelial cell sheets before and after transportation no longer adhere to the surface of the cell culture insert after peeling off (B and C). The cell sheets were detachable without microscopic alterations (B, phase-contrast microscopy) or macroscopic defects (C) after airplane transportation. There were no obvious changes between pre- and post-transportation samples in cell viability, the rate of pan-cytokeratin-positive cells, or the structure of the oral mucosal epithelial cell sheet (Supplemental Figure 2D-F). For evaluation of bacterial contamination during transportation, the culture supernatants in the primary cell sheet container were collected after transportation and subjected to sterility and *Mycoplasma* testing, and there were no infections. The scale bars represent 50 μm.

Supplemental Figure 2

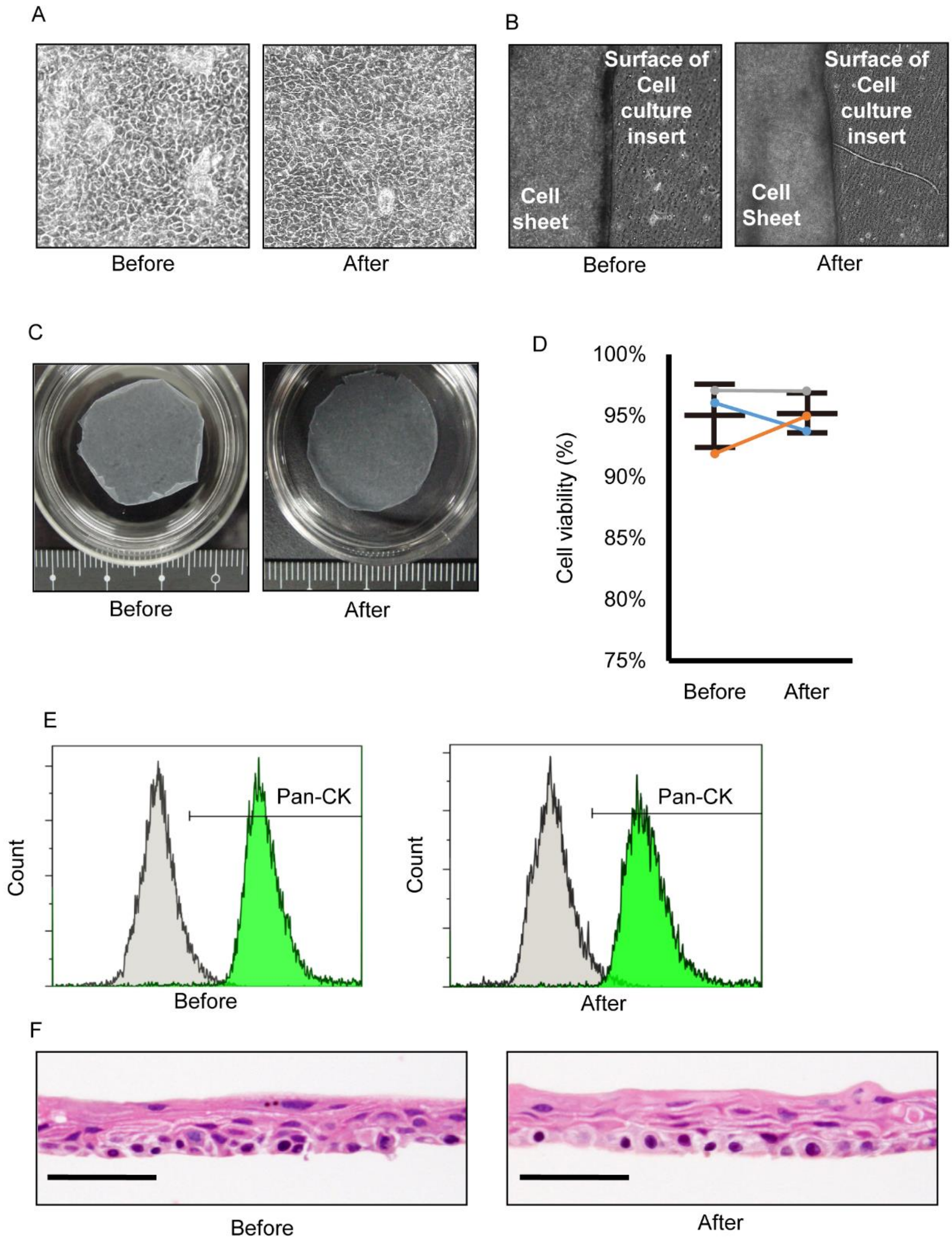

Supplemental Figure 3: Changes in plasma vascular endothelial growth factor (VEGF) levels, which were measured the day before ESD and cell sheet engraftment (Day 0), one day later (Day 1), and on Day 3, Day 7, and Day 28 (Supplemental Figure 3A). As the oral mucosal cell sheet fabrication was performed using autologous sera, we also confirmed the significant elevation of VEGF levels in the culture supernatant medium during cultivation by comparing the serum obtained 23 days prior to the cell sheet engraftment (Serum); on Day 0, the starting day of cell sheet fabrication; and on Day 15 of cultivation (Medium, Supplemental Figure 3B).

Supplemental Figure 3A

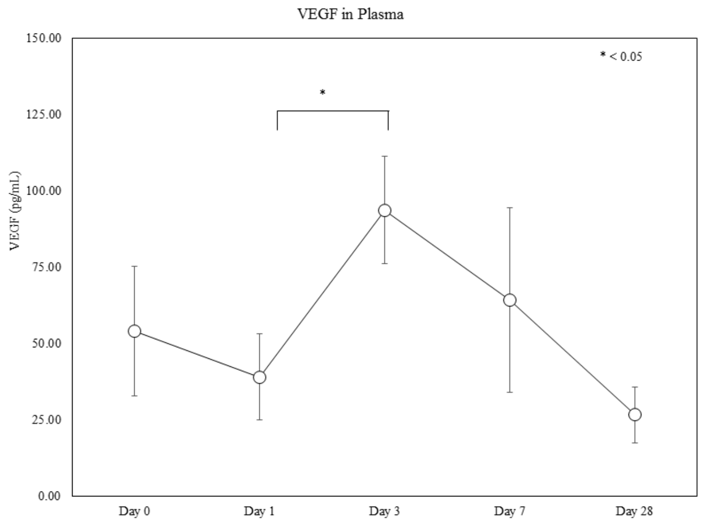

Supplemental figure 3B

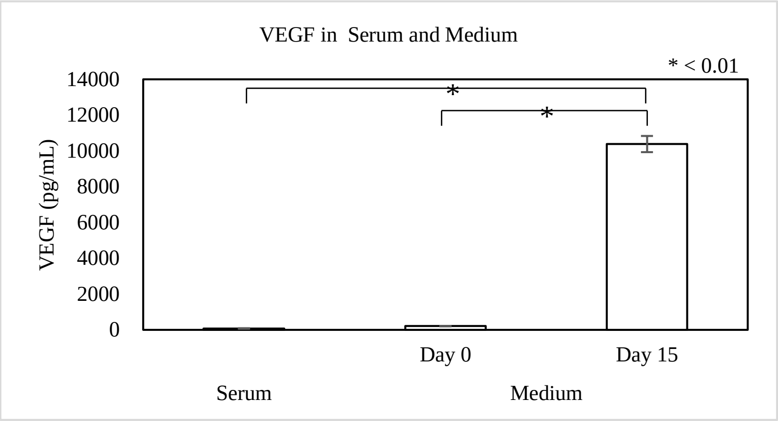

Supplemental Figure 4: In case #9, fully circumferential ESD was performed in an *en bloc* manner (A and B). Subsequently, 5 cell sheets that successfully transplanted were identified using standard and NBI endoscopy (C and D, respectively). Four weeks later, post-ESD stenosis (E) and related dysphagia occurred, which required one session of EBD on the 49th day. The post-ESD ulcer had healed without stenosis by the 70th day after transplantation (F).

Supplemental Figure 4

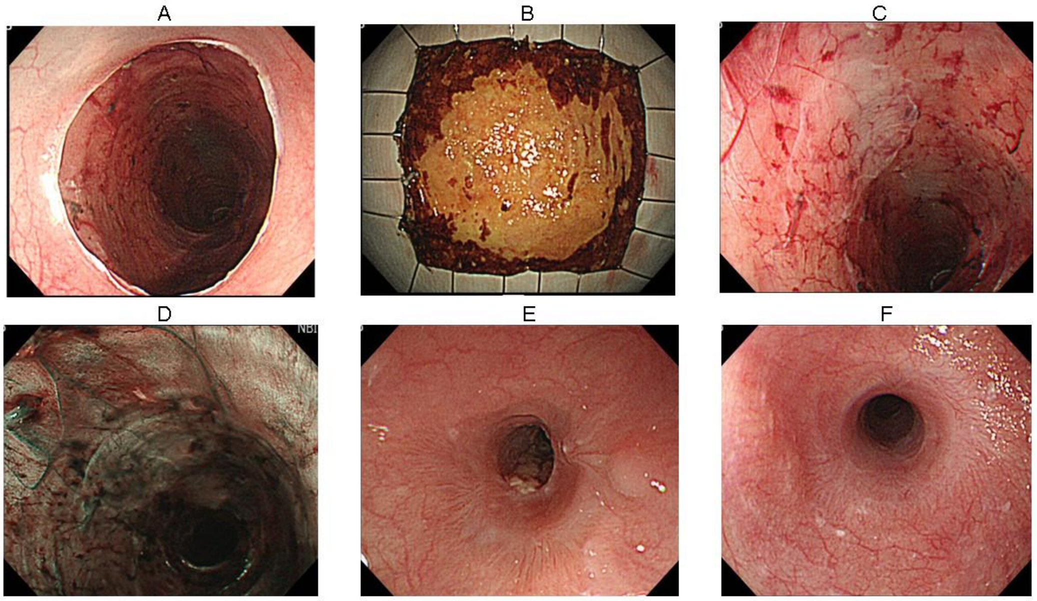

Supplemental Figure 5: In case #7, *en bloc* endoscopic resection of an ESCC lesion 73 mm in size was carried out, with the major axis of an ellipse of the resected specimen extending over nine-tenths of the luminal circumference. The post-ESD ulcer had already healed by Day 36 of follow-up endoscopy (A). Nevertheless, a biopsy was obtained from the engrafted centre of the cell sheet, and regenerative mucosa was histologically identified in the specimen (B). Immunohistochemical examinations showed sox2- and Ki-67-positive cells in the regenerative mucosa (C and D, respectively). The scale bars represent 100 μm.

Supplemental Figure 5

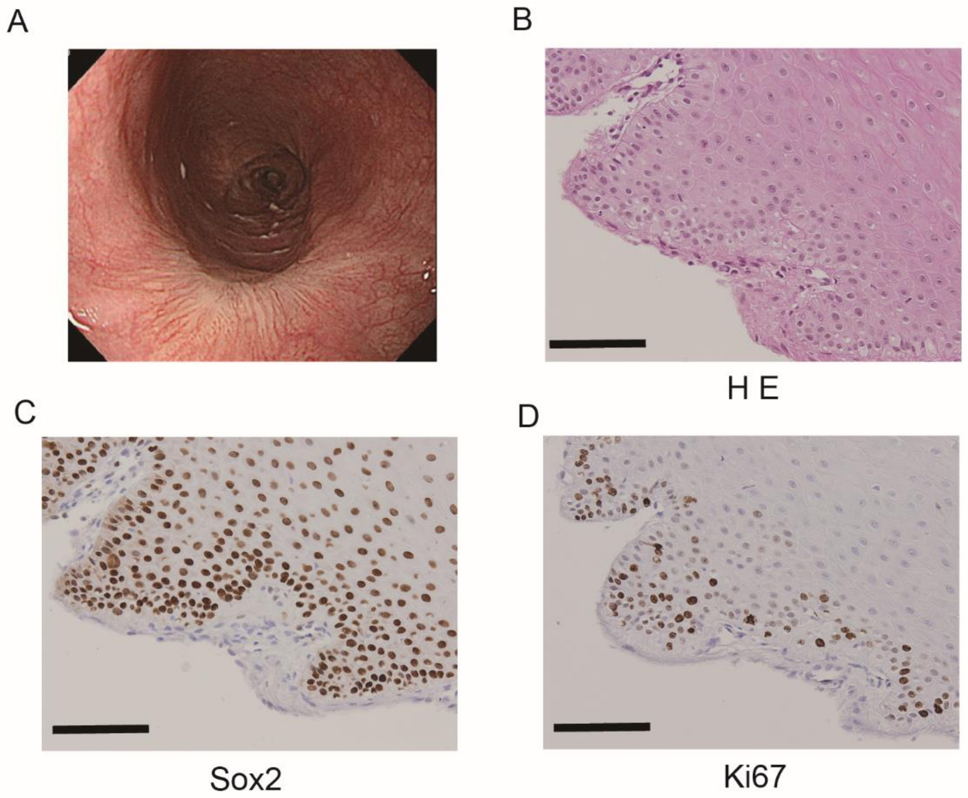

Clinical Research Protocol

Title		Oral epithelial cell sheets engraftment for esophageal strictures after endoscopic submucosal dissection of squamous cell carcinoma and airplane transportation
Research institute		
	Name	Nagasaki University Hospital
	Address	Sakamoto 1-7-1, Nagasaki 852-8523, Japan
	Telephone number	+81-95-819-7200 (Switchboard, Nagasaki University Hospital)
	Fax number	+81-95-819-7535 (Administration Division, Nagasaki University Hospital)
Head of research institute		
	Name	Shigeru Kohno
	Title	Director, Nagasaki University Hospital
Principal investigator		
	Department	Department of Surgery, Nagasaki University Graduate School of Biomedical Sciences
	Title	Professor
	Name	Susumu Eguchi
	Contact information TEL / FAX	TEL +81-95-819-7316 / FAX +81-95-819-7319
	E-mail	sueguchi@nagasaki-u.ac.jp
	Last degree earned	Doctor of Philosophy (medicine), Nagasaki University Graduate School
	Expertise	Gastroenterological surgery
Collaborative research institute		
	Name	Tokyo Women's Medical University
	Address	8-1 Kawadacho, Shinjuku-ku, Tokyo 162-8666, Japan
	Telephone number	+81-3-3353-8111
	FAX number	+81-3-5269-2367
Head of collaborative research institute		
	Name	Hiroshi Kasanuki
	Title	President, Tokyo Women's Medical University
Study objectives and goals		Endoscopic submucosal dissection (ESD) is a technique of endoscopic resection that allows direct dissection along the submucosal layer under the

	lesion. This technique enables *en bloc* removal of large superficial oesophageal cancer. However, oesophageal stenosis often occurs after ESD when a widespread lesion involves more than three-fourths of the luminal circumference. A study conducted by Ohki et al. of Tokyo Women's Medical University reported that oral mucosal tissues were taken from the buccal cavity of patients to fabricate autologous epithelial cell sheets on temperature-responsive culture dishes (cultured epithelial cell sheets) and that the cultured epithelial cell sheets were transplanted endoscopically onto the oesophageal ulcer after ESD. In their study, they found that transplantation of cultured epithelial cell sheets was safe and had the potential to prevent postoperative stenosis (Ohki T, et al. Molecular Gastrointestinal Medicine 6; 347, 2009; Ohki T et al. Gastroenterology. 2012). The current study is a clinical investigation in which cell sheets prepared by a different institution will be used. Thus, we believe that the study will have enormous significance in standardizing this therapeutic approach. Additionally, if this study can show more evidence that cultured epithelial cell sheets can be successfully transported and that the procedure is safe and effective, it may be designated an advanced medical treatment, leading to its widespread use.

Target disease	
Disease	Early oesophageal squamous cell carcinoma
Reason for selection	According to the clinical practice guidelines for oesophageal cancer published by the Japan Oesophageal Society, "the absolute indication for endoscopic resection is defined as carcinoma in situ or lesions limited to the mucosa that involve two-thirds or less of the luminal circumference." On the other hand, ESD enables curative resection of tumours regardless of their size. However, oesophageal stenosis often occurs after ESD when a widespread lesion involves more than three-fourths of the luminal circumference, resulting in a decreased QOL for the patient. This study aims to evaluate whether transplantation of cultured epithelial cell sheets can prevent post-ESD stricture in patients with early oesophageal cancer indicated for ESD and involving two-thirds or more of the luminal circumference. Additionally, we believe that promoting this therapeutic method at Nagasaki University is of great significance for the standardization of this therapeutic modality.

Inclusion criteria	All of the following inclusion criteria must be fulfilled at enrolment.<br>Inclusion criteria<br>1) Early oesophageal squamous cell carcinoma without metastases detected by imaging modalities including CT or endoscopic ultrasound.<br>2) Early oesophageal squamous cell carcinoma whose depth of invasion is limited to the carcinoma in situ or mucosa.<br>3) Early oesophageal squamous cell carcinoma involving two-thirds or more of the luminal circumference.<br>4) Men and women aged 20 to 85 years (inclusive) at the time when the study is explained to them to obtain their informed consent. The patient or his/her legal guardian must provide written informed consent for the patient to participate in the study.<br><br>Eligible patients must not meet any of the following exclusion criteria.<br>Exclusion criteria<br>1) Patients presenting any serious underlying disease (cardiac, renal or hepatic disease).<br>2) Patients with a malignant tumour other than oesophageal cancer.<br>3) Patients with an infectious disease (hepatitis B, hepatitis C, HIV, HTLV or syphilis).<br>4) Patients with any of the following haematological findings: WBC count <4,000/μL or ≥10,000/μL, platelet count <50,000/μL, AST (GOT) ≥100 IU/L, or ALT (GPT) ≥100 IU/L.<br>5) Patients with uncontrolled psychiatric disorders.<br>6) Pregnant women, lactating women, women of childbearing potential or women with planned pregnancy during the study period.<br>7) Patients with a disease of the oral mucosa at the tissue extraction site that makes tissue harvest impossible.<br>8) Patients who are considered by the investigator to be ineligible for the study owing to complications or other reasons.<br>9) Known drug hypersensitivity, prior use of medications or other factors that may affect the study results.<br>10) Radiotherapy from head to upper abdomen or a history of surgery that may affect ESD.
Human stem cells used for clinical study	
Type	Oral mucosal epithelial cells (autologous oral mucosal tissue derived)

	Method for extraction, preparation, transplantation or administration	(A) Preoperative tests<br>1) Prior to the conduct of any study treatment, the investigator will provide the patient with an adequate explanation orally, and the patient should voluntarily provide his/her informed consent in writing.<br>2) Preoperative tests—haematology, laboratory and blood coagulation tests—will be conducted as screening tests to confirm that there is no abnormality of the blood coagulation system.<br>3) Negative results in the patient should be confirmed for hepatitis B, hepatitis C, HIV, HTLV and syphilis.<br>(B) Preparation of the autologous sera<br>1) For autologous blood collection to obtain serum, determine the amount of blood to be taken, considering the number of cell sheets that the patient is expected to require. Confirm that the patient meets the requirements regarding general medical conditions and haemoglobin levels.<br>2) Collect 100-300 mL of blood more than 16 days before the scheduled surgery date (before extracting oral mucosal tissues) and take it to the CPC of Nagasaki University Hospital.<br>3) Transfer the blood to a 50-mL conical centrifuge tube and incubate it at 37°C for 1 or 2 hours to accelerate the blood clotting process.<br>4) Centrifuge the blood after clotting occurs, and collect the serum.<br>5) Centrifuge the blood again to collect more serum and filtrate using a syringe with a sterile filter.<br>6) Collect foreshots and serum in 15-mL conical centrifuge tubes and keep them in a freezer.<br>7) Transport the obtained serum to the Institute of Advanced Biomedical Engineering and Science, Tokyo Women's Medical University.<br>8) If the quantity of serum is insufficient, confirm that the patient's general medical condition is favourable, collect blood and repeat the procedure.<br>(C) Harvest of oral mucosal tissue and preparation of cultured epithelial cell sheets.<br>1) Set the date of the tissue harvest for approximately 16 days before the scheduled surgery date.<br>2) Inject a local anaesthetic and harvest oral mucosal tissue from the patient with a surgical knife or by punch biopsy according to the number of sheets required, taking into account the ulcer area estimated from the ESD resection

margin (approximately 20 mm^2/sheet, 4 - 6 sheets).

3) Perform compression haemostasis and suture the injured site.

4) Place the harvested tissue in PBS, then place the container on ice and transfer it to a biological safety cabinet.

5) Disinfect the tissue with Isodine and wash it twice in DMEM containing antibiotics.

6) Immerse the washed and disinfected tissue in a 50-mL conical centrifuge tube containing DMEM with antibiotics, and seal the container tightly, keeping it at approximately 4°C using cold compresses; then transport it to the CPC of Tokyo Women's Medical University by air and land.

<u>(D) Isolation and incubation of oral mucosal epithelial cells</u>

1) Place KCM (5% HS) in the temperature-responsive culture insert and preincubate it.

KCM: 71 v% D-MEM, 24 v% F12, 5 v% HS (autologous sera), 0.475 g/L L-glutamine, 5 µg/mL Humulin, 0.4 µg/mL Saxizon, 84 ng/mL (1 nM) CTL, 2 nM T3, 10 ng/mL EGF, 40 µg/mL gentian, and 0.141 µg/mL Fungizone.

2) Disinfect tissue fragments transferred to the CPC with Isodine and wash them twice in DMEM containing antibiotics.

3) Transfer the washed tissue fragments to dispase, immersing them with the epithelial side facing down; incubate them at 37°C for 2 to 4 hours or at 4 °C overnight.

4) Dissect the oral mucosal epithelium and incubate it with 0.25% trypsin at 37°C for 20 minutes.

5) Add KCM (5% HS), isolate cells by pipetting and remove unnecessary tissue to obtain a single-cell suspension; pass the suspension through a cell strainer.

6) Centrifuge at 1200 rpm for 5 minutes at 4°C, remove the supernatant and resuspend the cells in KCM.

7) Count the number of cells, and seed them on the temperature-responsive culture insert at a density of $4\text{-}8\times10^4$ cells/cm^2.

8) Change the medium at Days 5, 8, 10, 12, 13, 14, and 15, as well as after incubation on release day (Day 15).

<u>(E) Transportation of cultured epithelial cell sheets</u>

1) Perform quality control and verification for infectivity, cell markers and viability at the CPC of Tokyo Women's Medical University. Pack the samples in a special container for transportation (kept at approximately

		37°C), and transport them by air and land to Nagasaki University Hospital.<br>2) Take them out of the special container at the CPC of Nagasaki University Hospital and allow them to stand in the incubator (37°C, 5% CO_2) overnight.<br>(F) Transplantation of cultured epithelial cell sheets onto the oesophageal ulcer after ESD<br>1) Determine the lateral spread of oesophageal squamous cell carcinoma with iodine staining; mark the area.<br>2) Use sodium hyaluronate for local submucosal injection. Make an incision in the region outside the marking, dissect the submucosa and remove the lesion *en bloc*.<br>3) By the end of surgery, incubate the temperature-responsive culture dishes at 20°C for 30 minutes or longer.<br>4) Remove the KCM, wash the culture dishes three times with HBSS and collect the epithelial cell sheets from the temperature-responsive dishes.<br>5) Secure a working space for transferring the cultured epithelial cell sheets using oesophageal EMR (EEMR) tubes, commonly used for endoscopic mucosal resection of oesophageal cancer.<br>6) Transfer the cultured epithelial cell sheets to the post-ESD oesophageal ulcer endoscopically using a support that includes a transcription supporting film, taking care not to let the sheets adhere to the inner lid of the tube.<br>7) After transplanting (applying) cultured epithelial cell sheets onto the oesophageal ulcer, compress the sheets for approximately 10 minutes so that they adhere to the mucosa without sutures or adhesives.<br>(G) Postoperative assessment<br>1) Follow up the patient on an outpatient basis and examine him/her endoscopically every one to several weeks after the operation.<br>2) Follow up the patient for 12 to 14 weeks after the operation to check for any signs or symptoms of stricture (one-year follow-up).
Safety assessment		Presence/absence of adverse events (AEs), type, severity (mild, moderate, severe), safety, frequency and duration of AEs will be assessed. Observation and testing will be conducted at the time of surgery; 1, 2, 3, 4, 5, 12, 24, and 48 weeks after surgery; and at treatment discontinuation/end of treatment. A serious adverse event is determined on the basis of the following criteria regardless of the degree of symptoms:<br>1) Fatal<br>2) Life-threatening

	3) Requiring hospitalization as an inpatient or prolongation of existing hospitalization<br>4) Resulting in persistent or significant disability or incapacity<br>5) Congenital anomaly or birth defect<br>6) Other events that may affect the patient adversely and significantly
Reasons to conduct this clinical study	Ohki et al. of Tokyo Women's Medical University (Ohki T, et al. Molecular Gastrointestinal Medicine 6; 347, 2009; Ohki T et al. Gastroenterology. 2012) have already demonstrated that this modality of transplanting cultured epithelial cell sheets onto the post-ESD oesophageal ulcer is safe and effective in preventing the occurrence of oesophageal stricture. The oral cavity and oesophagus contain similar mucosal tissue consisting of stratified squamous epithelium; as the cell sheet is derived from autologous tissue, it is predicted that transplant rejection will not occur, and thus, engraftment of cell sheets is expected. Cultured epithelial cell sheets contain large amounts of stem cells and precursor cells, which help suppress inflammation and immune responses, facilitate healing of oesophageal ulcers and ultimately inhibit oesophageal stricture.<br>Tokyo Women's Medical University has a CPC (cell processing centre) in which cultured epithelial cell sheets are fabricated in accordance with GMP (Good Manufacturing Practice). In the preliminary study on the transportation of oral mucosa and cultured epithelial cell sheets, oral mucosa was extracted from healthy volunteers at Nagasaki University Hospital and transported to Tokyo Women's Medical University CPC, where cultured epithelial cell sheets were prepared, and then the sheets were transported back to Nagasaki University Hospital. The cell sheets to be used in this study were fabricated for a clinical study conducted by Tokyo Women's Medical University, and they met the all specifications when the sheets were successfully transplanted onto artificial ulcers. Moreover, when the cell sheets are transported to Nagasaki University Hospital, there should be no deviations from the specifications. On the basis of previous results, we deemed it possible to conduct the present study on "Application of Transplantable Autologous Oral Mucosal Epithelial Cell Sheets to the Oesophageal Ulcer After Endoscopic Submucosal Dissection (ESD) of Early Oesophageal Cancer" at Nagasaki University Hospital.
Study protocol	This study includes patients with early, non-metastatic oesophageal

	squamous cell carcinoma that involves two-thirds or more of the luminal circumference and whose depth of invasion is limited to a carcinoma in situ or in the mucosa. Eligible patients are those who meet all inclusion criteria and none of the exclusion criteria. The study period will be from January 2013 to June 2015. The target sample size is 10. After the last patient enrolment, a uniform survey will be conducted, and all patients will be followed up to 10 years.<br>This clinical study will be conducted in a way similar to the transplantation of cultured epithelial cell sheets onto post-ESD oesophageal ulcers at Tokyo Women's Medical University. After oral mucosa tissue is harvested and autologous blood is collected at Nagasaki University Hospital, the specimens will be transported to Tokyo Women's Medical University, where cultured epithelial cell sheets will be prepared returned to Nagasaki University Hospital to transplant onto post-ESD oesophageal ulcers. Specifically, these procedures will be conducted in accordance with the abovementioned "Method for harvesting, preparation, transplantation or administration".
Informed consent by the patients and other persons	
Procedure	The investigator will obtain written informed consent provided voluntarily by each patient participating in this study after full verbal explanation of the objective, method, procedure and risks involved in this study. The investigator shall give the patient or his/her family the opportunity to ask questions as well as enough time to determine whether to participate in the clinical study or not. The investigator must answer all the questions so that the patient will be satisfied.<br>The investigator, as well as each patient or his/her family, will date and sign (sign and seal) the Informed Consent Form and retain it. The Informed Consent Form should be provided in plain language and terms so that every patient and his/her family can understand it. The formats for the Informed Consent Form and the Consent Withdrawal Form are provided.<br>Any patient who cannot give consent on his/her own will not be eligible for this study.
Items to be explained<br><br>(Including benefits and potential	The investigator will prepare the Informed Consent Form (explanation document and consent form) and Consent Withdrawal Form. The explanation document should include at least the following items. The investigator should not intentionally include any description that may induce

	disadvantages for the patient)	a patient to participate in the study.<br>1) The objectives, goal and methods of the study.<br>2) The voluntary nature of participation. Refusing to participate will not affect the patient treatment in any way.<br>3) The ability of the patient to withdraw his/her consent at any time. Withdrawal from the study will not affect the patient's treatment in any way.<br>4) Alternative treatments.<br>5) Expected results and potential risks and unfavourable effects.<br>6) The protection of patients' personal data.<br>7) Actions after the completion of the study and publication of study results.<br>8) How to retain and use materials (documents) and the duration of retention (handling of materials (documents) after completion of the study)<br>9) Expenses and funding of the study.<br>10) Compensation.<br>11) Relevant organizations.<br>12) Study disclosure.<br>13) Provision of study results.<br>14) Intellectual property rights and other rights.<br>15) Contact information (name of research institutes, name of investigator, his/her title and contact information, etc.).<br>16) The patient's right to ask questions freely about this study.
In case of a clinical study that includes patients who cannot give consent on their own		
	Reasons that the study is necessary	Not applicable
	Policy for selecting a legally authorized representative	Not applicable
Actions to be taken in case that any serious event occurs in a patient, etc.		If any adverse event occurs, the investigator will provide appropriate urgent treatment, ensure the patient's safety and refer him/her to an expert to examine the cause. The investigator will take medical measures as needed to counteract any clinically significant adverse event during the study period or after study completion. Regarding AEs, the principal investigator will describe the type, onset date, severity (serious or not), clinical course and

	causal relationship with the clinical study in the Case Report Form. In particular, the principal investigator will perform a follow-up study for events whose causal relationship with the clinical study cannot be ruled out to a reasonable extent.<br>If any SAE occurs, the principal investigator will report it immediately to the Director of Nagasaki University Graduate School of Biomedical Sciences, who will report it to the Nagasaki University Ethics Committee. After the Director of Nagasaki University Graduate School of Biomedical Sciences receives the opinion from the Nagasaki University Ethics Committee, he/she will report it to the Minister of Health, Labour and Welfare to seek advice. The information to be reported will be shared with the President of Tokyo Women's Medical University through the collaborator at the same time.
Method for the follow-up study after completion of the study	The investigator will assess the complications and efficacy of this treatment at the time of the patient's regular hospital visits after completion of the study, enter the findings in the medical record and retain them as follow-up data. The follow-up period after the completion of the study will be 10 years, and the investigator will encourage the patient to visit the hospital regularly. The follow-up data obtained from the patient's regular hospital visits after completion of the study will not be included in the analysis.
Compensation related to the clinical study	
Compensation	Yes (circled)　　No
Describe in detail if there will be a compensation system	This study will include an insurance policy provided by a private insurance company as a compensation system in preparation for study-related injuries in the patient. For adverse health effects of this treatment that cause grade 2 disability or worse, as specified by the Relief System for Adverse Drug Reactions, medical expenses will be covered by the insurance plan. For other disabilities, this study will have a clinical study insurance policy from the same private insurance company. The details of compensation and indemnification will follow the provision of the insurance.
Method of personal data protection	
Method of linkable anonymization	Patient handling including data management and manufacturing control after obtaining consent from the patient will be conducted using linkable anonymizing patient ID code or registration number; a comparison table between the anonymized code and the patients' names will be stored securely in a locked place, along with all Informed Consent Forms including patient

		names.
	Other	The persons involved in the study must make the utmost effort to protect personal data. When the data centre refers to the medical institute to obtain information on a patient, the data centre will use a specific patient ID code managed by the investigator or subinvestigator or a registration number issued by the data centre. Monitors, auditors or staff from regulatory authorities who look through source documents with their own eyes must not leak the information obtained. When the study is published, the investigator must protect the personal data of the patients with great care so that the names of the patients will not be published.
